# Imaging Review of Neurofibromatosis: Helpful Aspects for Early Detection

**Published:** 2011-09-25

**Authors:** A. Hekmatnia, A. Ghazavi, M. J. Marashi Shooshtari, F. Hekmatnia, R. Basiratnia

**Affiliations:** 1 Associated Professor, Departmentof Radiology-Image Processing and Signal Research Center, Isfahan, Iran; 2 Resident of Radiology, Isfahan, Iran; 3 Assistant Professor, Department of Radiology, Isfahan University of Medical Sciences, Isfahan, Iran; 4 Medical Student, University of London, London, UK

**Keywords:** Neuro fibromatosis, Glioma, Schwannoma, Neurinoma

## Abstract

Neurofibromatosis (NF) is divided into two types, NF type 1 and NF type 2. Optic nerve gliomas have a high degree of association with NF type 1. NF 2, less commonly seen, is a complex of cutaneous and deep neural tumors. It is an autosomal dominant familial disorder in which CNS is affected in about 15% of the cases. Bilateral acoustic neuromas are pathognomonic of NF type 2 which may be associated with meningiomas or ependymomas.

Typical clinical manifestations of neurofibromatosis are cafe-au-lait spots and multiple cutaneous tumors. There is bone involvement as scoliosis, pseudoarthrosis of long bones, scalloping of vertebral bodies, abnormal rib tubulation and defective ossification of the skull. Extraskeletal manifestations of neurofibromatosis include optic nerve gliomas, pheochromocytoma, aneurysms of cerebral and renal arteries, acoustic neurilemmoma and superficial skin nodular neurofibromas.

Here, we intend to present images of several cases of neurofibromatosis with different patterns of body involvement.

## Introduction

Neuro fibromatosis (NF) is often noticed at birth or soon later. This manifests in several different ways. Typical clinical manifestations of NF are cafe-au-lait spots and multiple cutaneous tumors [1]. NF is divided into two types:

1. NF type 1 or Von Recklinghausen's disease: It affects 1 in 4000 of the population. It is an autosomal dominant familial disorder associated with a defect in the long arm of chromosome 17. The CNS is affected in about 15% of cases and the skull and spine are also frequently involved. CNS involvement of NF1 is associated with glial tumors, including optic nerve gliomas and cerebral and brainstem astrocytoma and also with anomalies of migration resulting in various types of dysplasia and heterotopia in the white matter. Hamartomas with no mass effect are frequently present, leading to areas of high signal on T2 MR studies. Generalized or focal cerebral atrophy may also be seen associated with mental retardation. Vascular dysplasias involving the terminal internal carotid or anterior or middle cerebral origins have also been described in NF1, as has the resulting moyamoya disease [2].

2. NF type 2: It is much less commonly seen, affecting only 1 in 50000 of the population. It is also inherited as an autosomal dominant condition, but is due to a defect of chromosome 22. The CNS lesions are quite different from those of NF1 and consist of bilateral acoustic neuromas which may be associated with schwannomas of other cranial nerves and meningiomas. In the spine, bony abnormalities are absent, but large bilateral neurofibromas may be seen. Spinal meningiomas as well as ependymoma of the cord or filum also occur [2].

Skeletal manifestations of NF1 include scoliosis, pseudoarthrosis of long bones (particularly the tibia) and hemihypertrophy. Scoliosis, often of short segment distribution, frequently occurs in the thoracic spine. In addition, numerous abnormalities arise related to the neural canal, including generalized dilatation with scalloping of vertebral bodies, internal meningoceles and dysraphic anomalies. Abnormal rib tubulation results in a ribbon-shaped appearance. A particularly common abnormality is defective ossification of the posterior superior wall of the orbit. Pseudarthrosis of the tibia is characterized by marked absorption of the fracture margins, so that they become pointed. Similar lesions may occur in the radius or clavicle [2].

Many extraskeletal manifestations of NF occur because of the neuroectodermal and mesodermal derivation of the tissue [3]. Gliomas of the optic nerves, pheochromocytomas, aneurysms of cerebral and renal arteries and acoustic neurilemmomas are well recognized. Similarly, the incidence of fibrous tumor of the bone is increased [4].

Study of neurofibromatosis imaging also has a significant role in prevention. As mentioned previously, neurofibromatosis is an inheritable disease. Therefore, early and correct diagnosis of the disease may be helpful in preventing disabilities in the next generation. Because sometimes the clinical manifestations of the disease are shown late or difficult to lead the clinician to the correct diagnosis, imaging may be a complementary limb in early diagnosis. For example, bilateral optic gliomas in an infant or an orbital defect in a neonate may guide the clinician and radiologist to the probable neurofibromatosis and early disease detection may prevent future disabilities of the patient in later life and subsequently transmitting the disease to the siblings.

Here, we intend to present images of several cases of NF with different patterns of body involvement.

## Cases Presentation

### Case one:

This case is a 20-year old girl with progressive loss of vision and cafe-au-lait spots on her skin.

**Fig. 1:**
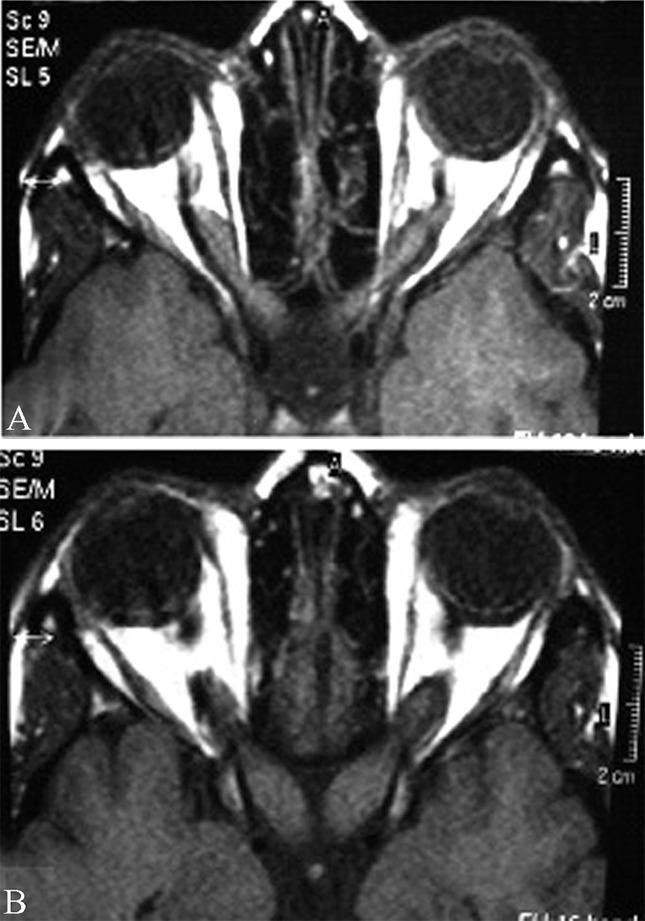
A 20-year old girl with progressive loss of vision and cafe-au-lait spots on her skin. A.Axial T1-weighted MR image of the orbits. B. Post-contrast axial T1-weighted MR image of the orbits.

In the axial T1-weighted MR image of the orbits, bilateral thickened optic nerves which were isointense with brain parenchyma were detected (Fig. 1A). In the post-contrast axial T1-weighted MR image, bilateral optic nerve gliomas without involvement of the chiasma were seen (Fig. 1B).

NF1 with bilateral optic nerve gliomas was the diagnosis. Histologically, optic nerve gliomas are juvenile pilocytic astrocytomas seen in up to 30% of all NF patients. 10% of all optic nerve gliomas are associated with NF[5].

### Case two:

She was a 23-year old girl with sensory-neural hearing loss.

**Fig. 2:**
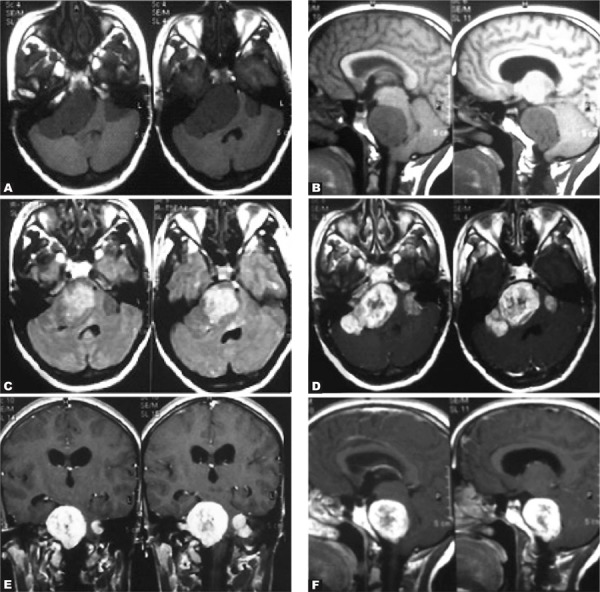
A 23-year-old girl with sensory-neural hearing loss. A and B. Axial and sagittal T1-weighted MR images of the brain. C. Axial FLAIR image. D-F. Axial, coronal and sagittal T1-weighted with contrast MR images.

Axial (Fig. 2A) and sagittal (Fig. 2B) T1-weighted MR images of the brain showed multiple masses with cystic degeneration in bilateral CP-angles as low signal intensity. Axial FLAIR image showed high signal intensity of the lesions (Fig. 2C). Axial (Fig. 2D), coronal (Fig. 2E) and sagittal T1-weighted with contrast MR images (Fig. 2F) showed heterogeneously contrast-enhancement of the lesions.

The masses were proved to be multiple acoustic neurinomas. One of them extended into the right internal acoustic canal as is shown on axial FLAIR image (Fig. 2C). Bilateral acoustic neurinomas are diagnostic for neurofibromatosis type 2 [5].

### Case three:

A 24-year old girl presented with bouts of headache and sensory-neural hearing loss.

**Fig. 3:**
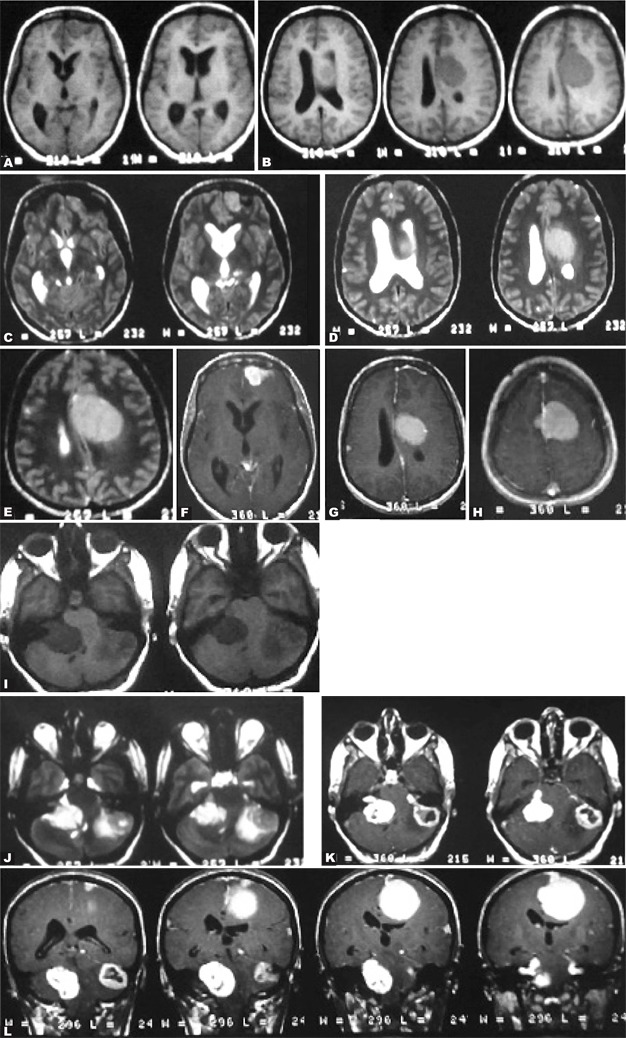
A 24-year-old girl with bouts of headache and sensory-neural hearing loss. A and B. Axial T1-weighted MR images of the brain. C-E. Axial T2-weighted MR images. F-H. Axial T1-weighted MR images with contrast. I. Axial T1-weighted MR images without contrast. J. Axial T2-weighted without contrast MR image of the CP-angles. K and L. Axial and coronal post-contrast T1-weighted MR images.

Axial T1-weighted MR images of the brain revealed an isosignal intensity meningioma in the left frontal lobe and another meningioma in the left parietal lobe with mid-line shift (Figs. 3A & B). Axial T2-weighted MR images of the brain revealed an isosignal intensity meningioma in the left frontal lobe and another meningioma in the left parietal lobe with mid-line shift (Figs. 3C-E). Axial T1-weighted MR images with contrast showed the same lesions isointense to brain cortex (Figs. 3F, G & H).

Axial T1-weighted MR images without contrast from CP-angles also show bilateral CP-angle masses as low signal intensity (Fig. 3I). Axial T2-weighted MR images of the CP-angles show bilateral high signal intensity masses of the CP-angles (Fig. 3J). Axial and coronal post-contrast T1-weighted MR images demonstrated heterogeneous enhancement of the lesions in favor of bilateral acoustic neurinomas (Figs. 3K & L).

Axial and coronal post-contrast T1-weighted MR images demonstrate severe homogeneous enhancement in meningiomas including dural tail (Figs. 3K & L).

As previously noted, bilateral acoustic neurinomas are diagnostic for neurofibromatosis. In NF2, there are some associations as meningiomas and ependymomas, the so-called MISME (Multiple Inherited Schwannomas, Meningiomas, Ependymomas).[6] This is a rare autosomal dominant syndrome characterized by propensity for developing multiple schwannomas, meningiomas and gliomas of ependymal derivation with an incidence of 1:50000 births. Meningiomas in this syndrome are located intraventricular in choroid plexus of trigone, parasagittal, sphenoid ridge, olfactory groove and along intracranial nerves. The symptomatic age of this syndrome is during the second/third decade of life.[5]

### Case four:

A 42-year old man with painful skin lumps on his body and weakness in his upper limbs.

**Fig. 4:**
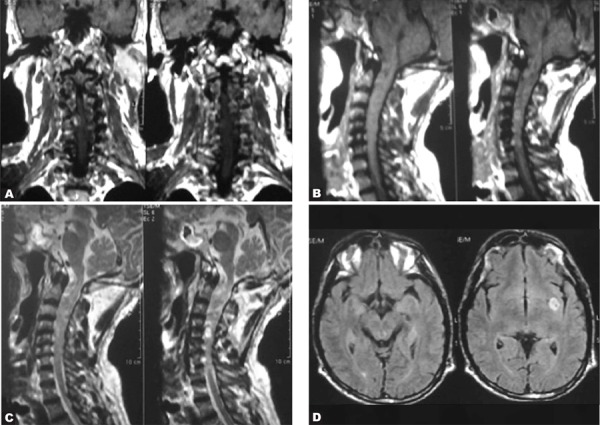
A 42-year-old man with painful skin lumps on his body and weakness in his upper limbs. A. Coronal T1-weighted MR image of the cervical cord B. Sagittal T1-weighted post contrast MR image of the cervical cord C. Sagittal T2-weighted MR image of the cervical cord D.Axial FLAIR image of the brain

Coronal T1-weighted MR image of the cervical cord demonstrated multiple ovoid subcutaneous tumors as low signal intensity (Fig. 4A). These are actually cutaneous neurofibromas. Coronal T1 (Fig. 4A), sagittal T1-weighted post contrast (Fig. 4B) and sagittal T2-weighted MR (Fig. 4C) of the cervical cord showed multiple neurofibromas in the cervical cord. Axial FLAIR MR image of the brain (Fig. 4D) revealed a heterogeneously high signal intra-parenchymal lesion on the left temporal lobe which is actually a hamartoma.

Hamartomas may be detected in up to 75 to 90 percent of the cases and are probably dysmyelinating lesions. They are located in the pons, basal ganglia (most commonly globus pallidus), thalamus and cerebellar white matter [5]. The above findings are compatible with NF1. In NF1, multiple lesions in the spinal column and cord may be seen including dysplastic neural foraminal enlargement and nerve sheath tumors which are actually neurofibromas. There may also be acquired meningocele which is a pulsion diverticulum from the spinal subarachnoid space. If the nerve sheath tumors are neurofibromas, the type of NF is one and if they are schwannomas, the type is two.

### Case five

A 25-year old man presented with weakness and paresthesia in his upper limbs.

**Fig. 5:**
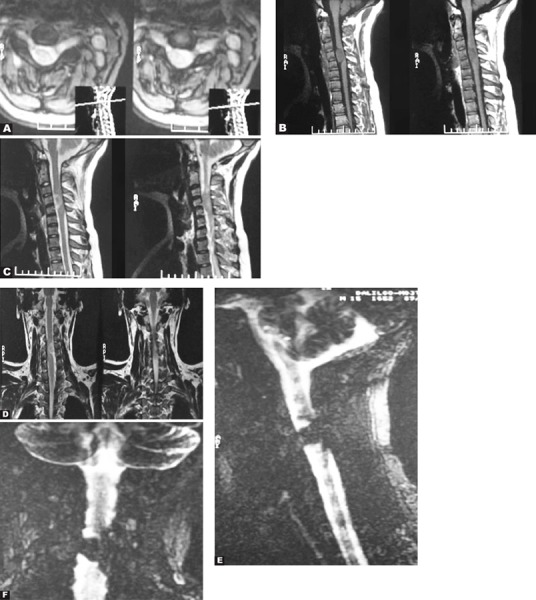
A 25-year-old man with weakness and paresthesia in his upper limbs. There is also an intra-dural extra-medullary mass with compressive effect on the adjacent cord. A. Axial T2-weighted MR image B. Sagittal T1-weighted without contrast C. Sagittal T2-weighted image D. Coronal T2-weighted image. E. MR Myelogram, sagittal view. F. MR Myelogram, coronal view.

Axial T2-weighted MR image of the cervical spinal column (Fig. 5A) revealed a bilateral dumbbellshaped high signal cervical cord mass which was proved to be neurofibroma after biopsy. There is also an intra-dural extra-medullary mass with compressive effect on the adjacent cord as can be seen on sagittal T1-weighted image without contrast (Fig. 5B), sagittal (Fig. 5C) and coronal T2-weighted images (Fig. 5D) and on the myelograms (Figs. 5E & F). The lesion was proved to be a neurofibroma after surgical resection.

The above findings are compatible with neurofibromatosis type one.

### Case six:

A 16-year old boy presented with deafness and skull deformity.

**Fig. 6:**
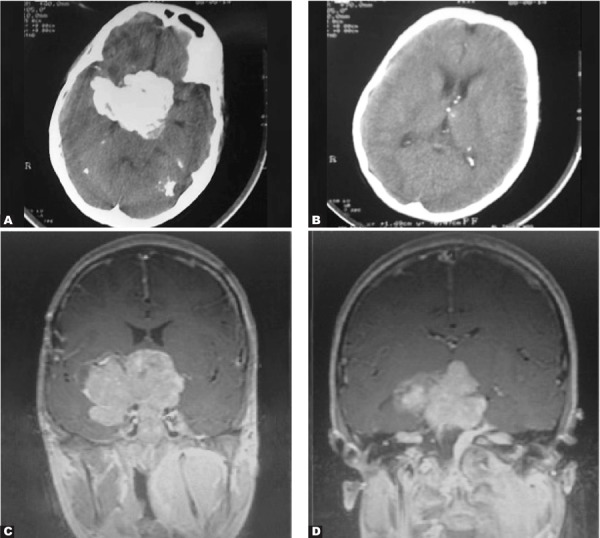
A 16-year-old boy with deafness and skull deformity. A and B. Axial post contrast CT scan C. Coronal post contrast T1-weighted image D.Coronal post Gd injection

Axial post contrast CT scan image of the brain showed multiple extra-axial coarse calcifications which were proved to be meningiomas including intraventricular meningiomas (Figs. 6A & B). Coronal post-contrast T1-weighted MR image showed the same large calcified mass on CT which is contrast enhanced and well defined with a mass effect in this image (Fig. 6C). Coronal post Gd injection MR image showed bilateral acoustic schwannomas as enhanceable lesions in CPangles (Fig. 6D).

Bilateral acoustic neurinomas and multiple meningiomas are diagnostic for NF2.

### Case seven:

A 30-year old woman complained of abdominal pain.

**Fig. 7:**
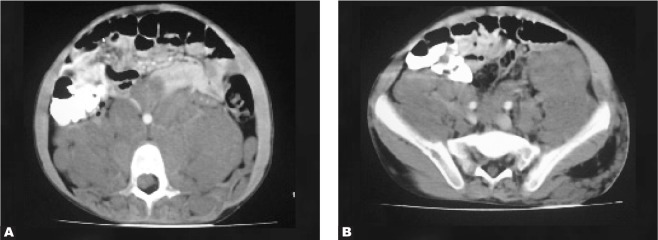
A 30-year old woman with abdominal pain. A. Axial CTscan with IV and oral contrast B. Axial CTscan with IV and oral contrast

CT scan of the abdominopelvis with IV and oral contrast revealed a large retroperitoneal lobulated nonenhanced and homogeneous mass with encasement of the aorta and extension into the neural foramina. There is also neural foraminal widening of the lumbar vertebra. The para-spinal muscles contained multiple small non-enhanced, homogeneous masses. The masses were proved to be neurofibromas from the neural foramina to the retroperitoneal space (plexiform neurofibromatosis- type one) and from the para-spinal muscles (Fig. 7A). On the lower slice of the same CT scan, multiple subcutaneous neurofibromas and the extension of the retroperitoneal neurofibroma to the pelvic cavity were demonstrated (Fig. 7B).

### Case eight:

A 19-year-old man complained of a 6-month pain on the left side of the thoraco-lumbar area.

**Fig. 8:**
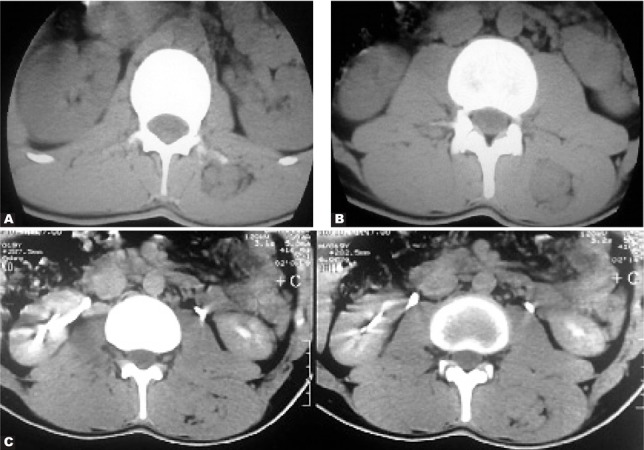
A 19-year-old man with 6-month pain on the left side of the thoraco-lumbar area. A and B.Axial CTscan without IV contrast C.Axial CT scan after contrast injection

Axial CT scan without IV contrast showed several ovoid contiguous masses inside the left-side quadratus lumborum muscle. These masses extend downward and may be detected in two axial consecutive sections. The masses were isodense to the adjacent muscle(Figs. 8.A & B).

After contrast injection, the axial CT scan at the level of the kidneys reveals the mentioned masses, which are again isodense to the adjacent muscles (Fig. 8C).

The pathological examination demonstrated that the resected masses were neurofibromas. After scrupulous physical examination, the patient had cafe-au-lait spots on his skin (NF1).

### Case nine:

A 13-year old boy with apparented deformity of the spinal column and recent dyspnea.

**Fig. 9:**
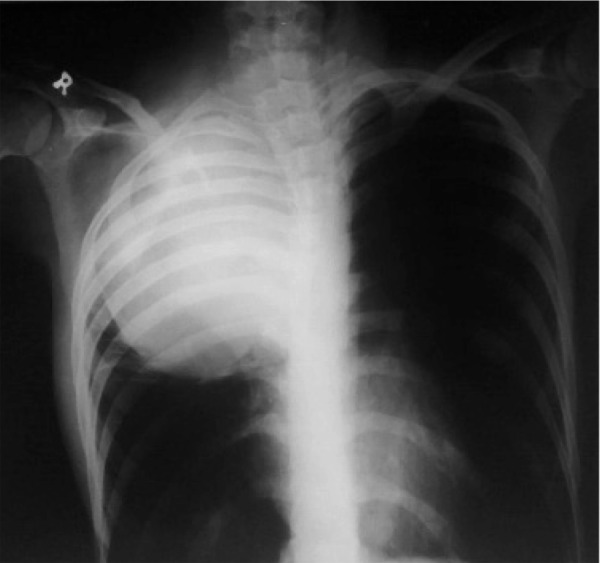
A 13-year-old boy with apparent deformity of the spinal column and recent dyspnea. PA chest X-ray.

In the PA chest X-ray, there is scoliosis in the thoracic spinal column and a soft tissue mass in the upper posterior mediastinum (Fig. 9) which was proved to be a neurofibroma after biopsy. With careful skin examination, cafe-au-lait spots were also found (NF1).

### Case ten:

A fifteen-year old boy presented with swelling of the right lower neck.

**Fig. 10:**
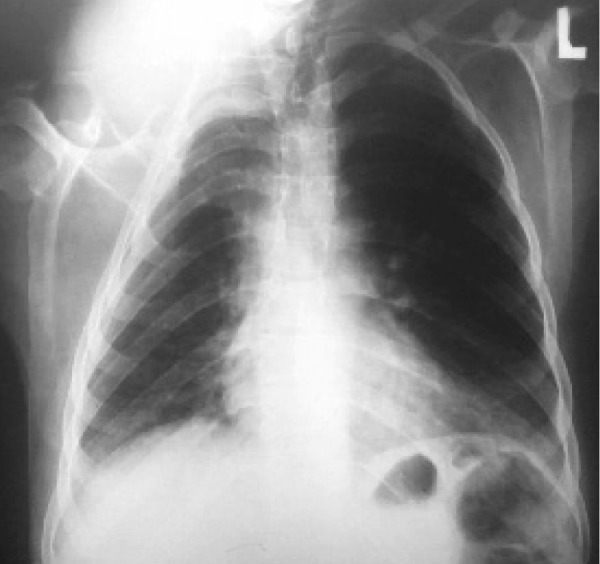
A fifteen-year-old boy with swelling of the right lower neck. PA chest X-ray

The postero-anterior chest radiograph revealed a subcutaneous soft tissue mass in the right supraclavicular area with extension to the lower neck. The mass had pressure effect on the trachea. There is also short-length scoliosis of the upper thoracic spinal column (Fig. 10). The mass was proved to be a large neurofibroma after surgical resection (NF1).

### Case eleven:

A 20-year old boy presented with deformity of the chest.

**Fig. 11:**
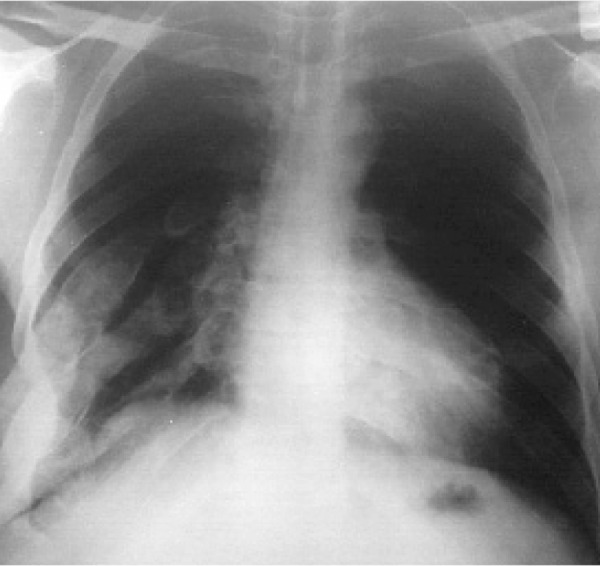
A 20-year-old boy with deformity of the chest.PA chest X-ray

The plain PA chest radiograph showed tubulation of at least four ribs in the right lower thoracic area (Fig. 11). Rib tubulation is another feature of NF1 which is actually intra-costal neurofibromas causing rib deformity.

### Case twelve:

An 18-year-old man presented with apparent leg deformity and cafe-au-lait spots.

**Fig. 12:**
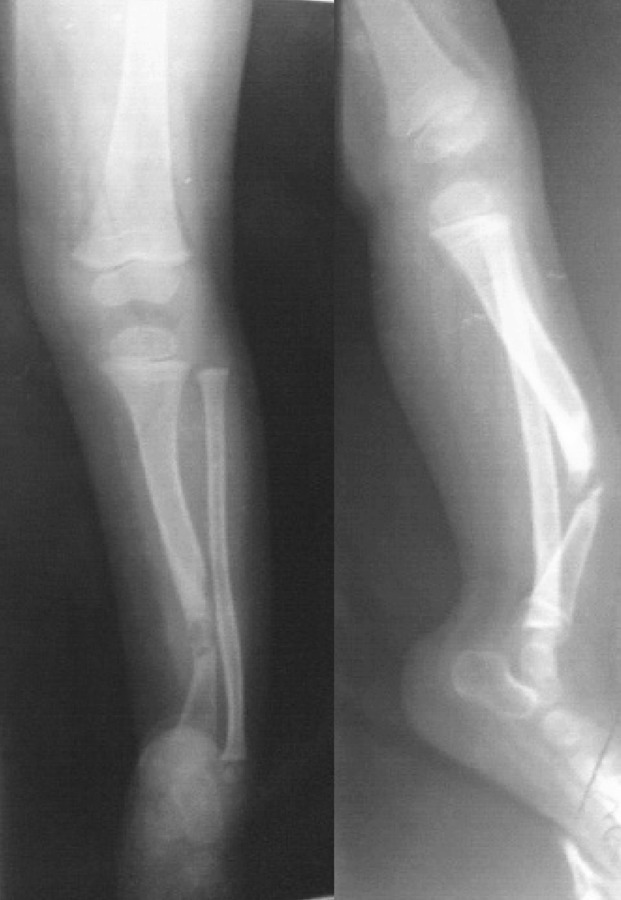
An 18-year-old man with apparent leg deformity and cafe-au-lait spots. Lateral and AP views of the lower extremity.

AP and lateral views of the leg showed pseudarthrosis of the tibia (Fig. 12) indicating NF1.

### Case thirteen:

A 12-year-old girl presented with cafe-au-lait spots and apparent deformity in the forearm.

**Fig. 13:**
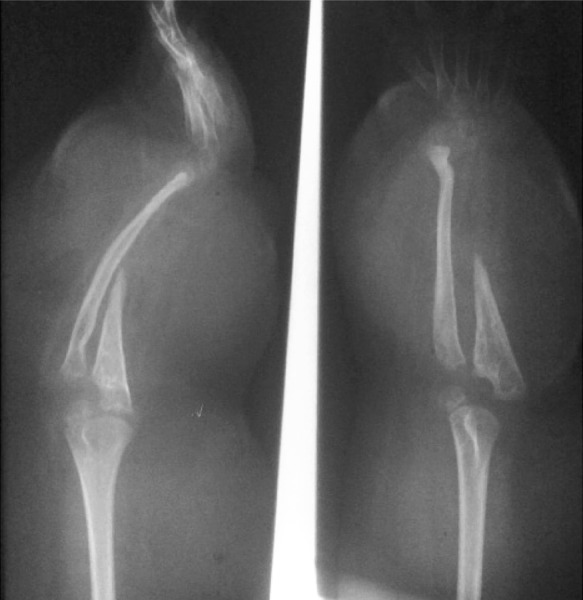
A 12-year old girl with cafe-au-lait spots and apparent deformity in the forearm. PA and Lateral views of the forearm and hand.

PA and lateral views of the forearm and hand showed severe pseudarthrosis of the radius and ulna resulting in marked absorption of the fracture margins (Fig. 13). The surrounding soft tissue swelling is due to large-sized neurofibromas compatible with NF1.

### Case fourteen:

A 40-year-old man presented with swelling and pain in the left ankle.

**Fig. 14:**
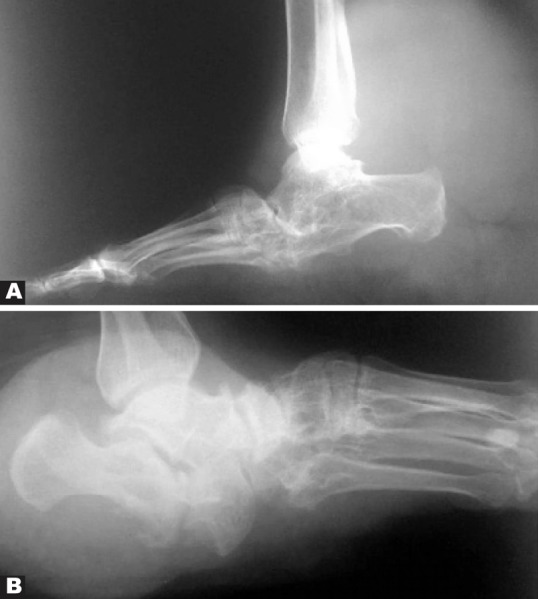
A. A 40-year-old man with swelling and pain in his left ankle. Lateral X ray of the left ankle. B. Lateral X ray of the ankle in another case of neurofibromatosis type one.

Lateral view of the ankle showed a large-sized tumor at the back of the ankle which was proved to be neurofibroma. Other smaller soft tissue neurofibromas may also be detected. Pseudoarthrosis of the talo-calcaneal joint is also seen (Fig. 14A). Another different case of neurofibroma of the ankle area in a neurofibromatosis type one patient has been shown in figure 14B.

### Case fifteen:

A 17-year old girl presented with chronic left foot swelling.

**Fig. 15:**
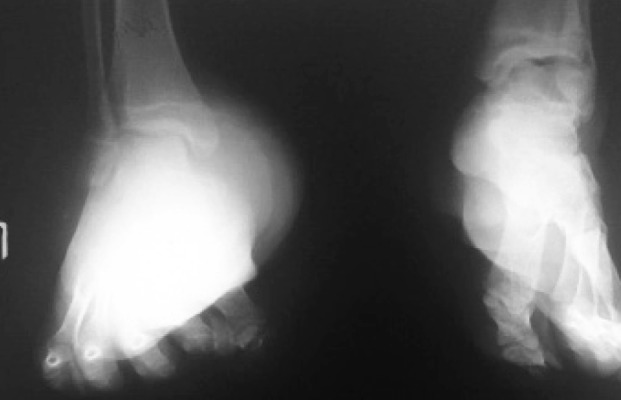
A 17-year-old girl with chronic left foot swelling. AP and oblique views of the foot.

AP and oblique X ray views of the foot showed a large soft tissue swelling on her left foot similar to previous case including deformity of tubular bones of metatarsals and phalanges (Fig. 15). The mass was confirmed to be neurofibroma after surgical resection. There were also several subcutaneous neurofibromas in other parts of her body (NF1).

### Case sixteen:

An infant presented with hand deformity.

**Fig. 16:**
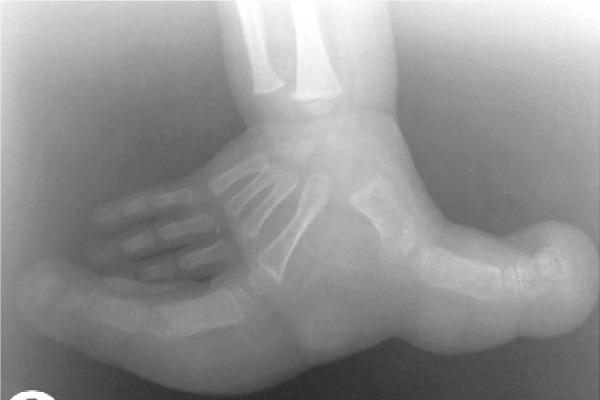
An infant with hand deformity. AP X ray.

AP plain radiograph of the hand revealed deformity as local gigantism of first and second fingers (Fig. 16) which is due to NF1. Local gigantism is another feature of NF. There were subcutaneous neurofibromas in other parts of his body.

### Case seventeen:

A 25-year-old woman presented with swelling of fingers.

**Fig. 17:**
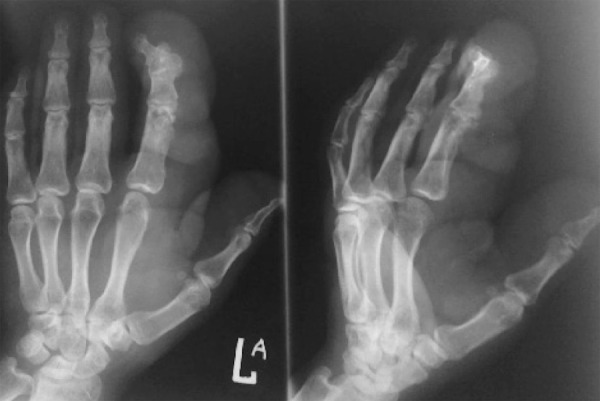
A 25-year-old woman with swelling of fingers. AP and oblique views of the hand.

The AP and oblique views of the left hand revealed soft tissue swelling of the thumb and index finger (Fig. 17) due to multiple masses which were proved to be neurofibromas (NF1). There is also deformity of interphalangeal joints of the index finger.

## Discussion

Neurofibromatosis has various imaging manifestations which are different in each of type one and two neurofibromatoses. All imaging modalities such as plain radiographies, CT scan and MRI may demonstrate various aspects of neurofibromatosis imaging and in a position to initially suspect NF, the radiologist should be aware of the manifestations. One should also be aware of the accompanying involvements of NF such as optic glioma, meningiomas, ependymomas and pheochromocytomas.

NF may involve different parts of the body such as the brain, spinal cord and exiting nerves, bones, soft tissue, kidneys and even the mediastinum. Therefore, evaluation of other parts, if in doubt, is necessary. Involvement of each part of the body may also have different imaging manifestations; for example bone involvement in NF may be in the form of scoliosis, pseudarthrosis, bony defect, posterior scalloping of the vertebral bodies and tubulation of the ribs.
